# Diabetes

**Published:** 1858-12

**Authors:** W. Matthews

**Affiliations:** Nicholsonville, Putnam Co., Ind.


ARTICLE IV.

### DIABETES.

**(A Paper read before the Hendricks County Medical Society, at its July meeting.)**

BY W. MATTHEWS, M.D., OF NICHOLSONVILLE, PUTNAM CO., IND.

MR. PRESIDENT AND GENTLEMEN OF THE SOCIETY,—At the last meeting of this society I was requested to prepare a paper to be read at this time. Not feeling at liberty to disregard the request thus kindly extended to me, I have selected for a subject that ancient, yet very imperfectly understood, disease known as diabetes.

Diabetes is a disease of very considerable interest to the medical practitioner. Being a very rare affection, this circumstance of itself renders a cure of it a matter of interest. In addition to its rarity, its illy defined pathology, and almost uniform fatality, give additional gravity to the subject. That the disease is a rare one every experienced physician must admit. A tolerably full practice of nearly sixteen years has furnished me with but a single well-marked case; and I have conversed with a number of practitioners of considerable experience, who never saw an instance of the disease. And, indeed, our authors bear evidence to the same general purport, though it is to be remarked that some of the older writers speak of the disease as somewhat common—a circumstance to which I shall advert presently.

As to the pathology of the affection I would only remark in this place, that it is a matter quite unsettled; and agreeable to the best late writers, very few, if any, cases of the disease ever get entirely well.

Now, as I propose speaking of the disease somewhat in detail, let us in the first place narrate its most prominent symptoms. It was known to the ancient Roman physicians. The name appears to have been derived from two Greek words, signifying "to pass through"—from the great quantity of aqueous fluid, I suppose, which passes through the kidneys and bladder. "Diabetes," says Eberle, who is in general a correct describer of disease, "usually makes its appearance in a very gradual manner, although, in some instances, it comes on suddenly, with slight chills and febrile commotions. When its invasion is gradual, it is generally attended from the first with various indications of a disordered condition of the digestive organs—such as variable appetite, acid eructations, occasional nausea and vomiting. The quantity of urine discharged in this affection," he continues, "is almost always extremely great, and in some instances truly enormous. I have seen," says he, "two cases, in which from twelve to fifteen pints of urine were discharged in the course of twenty-four hours, for several weeks. The urine in this disease," says the same author, "is generally of a pale straw color, approaching sometimes to a shade of green. The urine gives off a faint odor, resembling somewhat that of fresh animal broth, and its taste is sweet. It contains very little or no urea, and it passes readily into the vinous fermentation." I continue to quote from the same authority. "The constitutional symptoms which attend this disease are—urgent thirst; craving appetite; dry skin; a distressing sense of weight and uneasiness in the stomach after taking food; dry and parched mouth; white, foul, sometimes clean, and red tongue; wasting of the flesh; a feeling of languor and aversion to exercise; debility; pain and weakness in the loins; irregular action of the bowels, being most commonly costive," etc., etc.

With this sketch of the symptoms of diabetes, I pass to the consideration of its causes and pathology, only remarking that the most prominent symptoms are dry tongue, dry mouth, rapid emaciation, and the discharge of an inordinate quantity of urine, more or less loaded with saccharine matter. To constitute a case of genuine diabetes, the urine must contain saccharine substances; and I shall, therefore, in the present paper, confine my remarks to saccharine diabetes.

"We know but little about the causes of diabetes," says Dr. Watson; and apparently to avoid the investigation, so fruitless to all his predecessors, he adds, that, in "those afflicted with it, a predisposition to it probably existed." But in what this predisposition consists we are left to conjecture. If Dr. Watson is correct in his notion that the disease is frequently hereditary, and he mentions several cases which appear to support such a view, I would ask, in what does the predisposition consist? Does the hereditary vice consist in primary organic or functional disorder of the organs of assimilation in, to use other words, hereditary indigestion? "It occasionally seems to be," says he, "produced at once, by the operation of some exciting cause, such as exposure of the body to cold," etc. Now the question is, how does cold produce diabetes? "Distress and anxiety of mind are held," says the same authority—and justly, he thinks—"to be among the predisposing causes." Distress and anxiety of mind, if we admit them to be predisposing causes, must have their primary seat in the brain; and "cold," too, not unfrequently gives rise to nervous affections.

Thus may not the disease in question properly be referred to the nervous system as its primary seat? It is well known that neurotic disorders, of all others, are most often transmissible from generation to generation. Apoplexy, epilepsy, hysteria and chorea, are familiar instances of the truth of this assertion.

But there are better grounds than those just stated for referring the primary seat of this disorder to the brain, or rather nervous system. When we turn back to the older writers upon the subject under consideration, they tell us that diabetes has its seat in the kidneys; and to fortify their position, we are told by them that autopsic inspection bears testimony to this view; for, say they, while it very often happens that no traces of disease can be found in the whole digestive apparatus, the kidneys are always more or less altered from their healthy appearance. More recent and, as is presumed, better informed pathologists, however, while they admit the fact that the kidneys, during the progress of diabetes, undergo very manifest structural alterations, they contend that those alterations are solely due to excess of function, and not to primary disease—that is, that the kidneys have simply been overworked, and, as a consequence, are more or less hypertrophied. And such, no doubt, is the truth of the matter. But it is to be remarked, that while the more recent writers succeed in destroying the kidney theory of diabetes, they have failed to establish a more rational solution of the difficulty. They tell us, to be sure, that the vice is to be looked for as a simple functional difficulty in the chylopoietic organs; that the fault lies in some obscure variation in the assimilative process. But dissection fails to exhibit any material changes in any of the structures immediately concerned in eliminating the food; and we are again left to uncertain conjecture.

There is, to a certain extent, at least, a solecism involved in speaking of simple functional disease. It is almost equivalent to talking about a disease without a cause. A watch may run continuously but irregularly; but that irregularity, it must be confessed, depends upon the variable application of certain physical laws or forces. If these laws and forces were equitably supplied, the watch would move with the exactitude of the heavenly bodies in their spheres. So of the human mechanism: supply it with all the physical conditions of health, and we should have no functional disorders to quibble about.

I am, therefore, for the reasons above suggested, strongly inclined to doubt the propriety of dividing diseases into organic and functional, merely because we are frequently unable to behold, with our imperfect means of investigation, changes of structure to which to refer them. In the present instance, then, I am led to look beyond the altered condition of the kidneys for the cause of disease. I go to the viscera—the stomach and bowels, and their co-laborers in the work of assimilation. But here I find no evidence of primary disease. All the organs of this system are, apparently, in a state of physical integrity not incompatible with perfect health. But there is irregularity of function. The inner guard which stands sentry at the entrance of the very fountain of life has failed to perform its duty, and foreign matters have found their way into the circulatory fluid. Sugar, a natural enemy to the life-forces, has found its way into the mass of fluid that is sweeping with fearful speed through every ramification of the body, supplying this tissue with appropriate nutriment, and removing from that hurtful and pestiferous waste matters—these latter being presented to the various outlets of the body, and through their appropriate channels expelled therefrom. Thus it is that the sugar, surreptitiously, so to speak, introduced from without, a blood poison, upon reaching the kidneys, is permitted to escape; but in finding its way out through these organs, the foreign saccharine substance acts upon them as a powerful diuretic, and the consequence is, that the urine is poured forth in inordinate quantities, greatly diluted, and holding in solution the peculiar sweet substance known as diabetic sugar. It is proper here to state, that the chemical relations of the urine itself are not necessarily greatly varied from that of health, except in dilution, and the one foreign compound, diabetic sugar—glucose. I am aware that this statement may be called in question, but it is presumed to be mainly correct.

The sugar, therefore, in the urine is to be regarded as the result of a blood poison; and its introduction into that fluid—the blood—is to be attributed, not to primary disorder of the kidneys, nor yet of the stomach and bowels, but of the nerves of organic life which supply the chylopoietic viscera. And we are not to look to the extremities of these nerves for the source of the trouble, but we are to go to the source of the nerves themselves—the brain—to find it.

Thus, I shall contend that diabetes is neurotic in its nature, and that its primary seat is to be sought for in the brain, and that therapeutical agents used in its remedial management should be addressed, at least in part, to the primary lesion.

Now then, I shall proceed to offer a few remarks in support of the neurotic origin of diabetes, and I beg leave to say, that if I produce but one proof, plain, clear and indisputable, I think the argument will be entitled to respect at least, since such a proof has, so far as I know, never been furnished the profession by any of the numerous theories in popular vogue.

"M. Bernard found," says Dr. Wood, "that by irritating the the olivary bodies of the medula oblongata, he could at any time increase the production of sugar, and cause its appearance in the urine." That is, by the irritation of these bodies the digestive process is so far removed from the control of the vital laws, as to permit the supremacy of the chemical laws, and in this manner allow the starch, etc., contained in the food, to undergo transformation into sugar, and the result of this transformation, being absorbed into the blood, is expelled unchanged along with the urine.

Again, Dr. Prout remarks, that "so far as his experience goes, carbuncle is always accompanied by diabetes." May not, let me ask the question, carbuncle frequently produce irritation of the medullary bodies in question? Indeed, we know that carbuncles, located on the superior portions of the body, not unfrequently destroy life by cerebral disease. And I therefore infer that carbuncle, so commonly met with in diabetic patients, should be, in some instances at least, looked upon as a cause rather than a complication of the disease. But more to the point are the two following cases, the substance of which I extract from the June number of the *Medical News and Library*:

A woman, recently under the care of Dr. Todd, of King's College Hospital, after having received a severe injury of the head, became diabetic. She had hemiplegia, rigidity of the right arm, etc., and died. Dr. Goolden, says the same report, treated a case some years ago, in the course of which, the patient, a man who had received a very severe stroke upon the occiput, became diabetic.

Now, the evidence just offered establishes very clearly to my mind, at least, the cerebral origin of diabetes. It might, indeed, be very difficult to point out the exact pathological nature of the affection; but we are by no means to be astonished at this. We know that epilepsy, catalepsy, chorea, hysteria, etc., are nervous disorders, but no one has, I believe, furnished the profession with the special pathology of either of these affections; and the same may be said of several other neurotic affections. In certain forms of paralysis, for example, the exact or special pathology is not well understood. In the white softening of the brain it is inferred, I believe, that there is loss of substance, an atrophy; and this inference is strengthened from the fact that patients laboring under the affection are frequently restored to health by a tonic plan of treatment. The organ is put upon a better regimen, a nutrition richer in the elements of its growth, and as its bulk is increased, the manifestations of disease are, so to speak, pushed aside. So of chorea; sulphate of zinc cures certain cases of chorea by somehow aiding the nutritive process concerned in supplying the nervous wastes.

For the purpose of illustrating the present subject more fully, especially in a practical point of view, I will here introduce the substantial history of a single case, not attempting to descend to minutiæ.

On the 6th of April last, I was requested to examine Mrs. K., a lady about sixty years of age. I had occasionally seen her before, and was now somewhat surprised to see her so thin and shrunken in appearance. I remembered her only as a plump, plethoric, hearty-looking old lady, who would probably have weighed 160 pounds. Her appearance was now that of a person who had been reduced to death's-door by starvation. The surface of the body was dry and shrunken, the adipose matter gone, and the muscles greatly attenuated. Her manner was unsteady, hurried and agitated. The pulse was not much unnatural, perhaps rather small and hard. She was free from cough, and suffered no acute pain; even those neuralgic pains, so commonly complained of in the left side of certain females, and from which this old lady had suffered more or less for many years past, had given her no uneasiness for two or three months. Her mouth was dry and the tongue covered with a white coating, and the bowels were constipated. She had great thirst—insatiable and voracious appetite, which she could find no means of satisfying. And for two or three months she had passed, according to her own statement, from one to two gallons of a pale straw-colored sizy urine every twenty-four hours, and daily emaciation had been going slowly on during the whole time. She had had but little treatment, and that without any permanent advantage to her.

This was a case, as I then inferred, and I imagine correctly, of diabetes mellitus. Not having the necessary means of testing the urine by chemical reagents, I put a couple of ounces in a vial and added to it a small quantity of well fermented yeast, and in the course of a few hours the whole was in an active state of vinous fermentation—a circumstance indicating beyond the possibility of reasonable doubt the presence of sugar.

She was advised to eat nothing containing sugar, and to avoid, as far as possible, as articles of diet, those substances containing starch. Meat, eggs, milk, butter, cheese, and the leguminous garden vegetables, were recommended for food, with small quantities of stale bread. She was also advised to take creosote and opium three times daily, and a powder of sulph. cinchonia and carb. ferri about the middle of the fore and afternoon each day. She was also allowed as much brandy as she might desire, and her bowels were to be regulated by stimulating enemata.

Under this management the old lady was more comfortable, and the quantity of urine was perceptibly diminished. But after being kept upon the treatment a good many days, I was satisfied that the emaciation was not arrested, nor were the physical appearances of the urine at all altered for the better. About this time she was put upon an infusion of rennet, I having obtained the knowledge of its reputed virtues in this class of cases from our distinguished friend and the friend of science, D. Hutchinson, M.D., ex-president of this society. But although used for a considerable length of time, was unable to perceive that the patient derived any benefit from its employment; besides, the solution was exceedingly offensive to her.

At this time, after the old lady had been under treatment about five weeks, her condition was apparently hopeless; she was growing thinner and more feeble daily, though her appetite remained still morbidly good. Her skin and mouth remained dry, and she was agitated by nervous tremors. I now as a last and almost forlorn hope resolved to put her upon the following prescription:

℞ Pulv. ipecac., ʒ i.

Nit. argent.,

Pulv. opii, each, grs. xv.

Mix, and form into sixty pills; one to be taken three times a day, one hour before eating.

Under the employment of this prescription the patient was better on the second day, and very manifestly so on the fourth. In one week the quantity of urine was diminished very nearly within normal limits, and its physical *appearance* was that of healthy urine. The mouth was moist and the thirst very moderate, the skin was covered with a healthy, warm perspiration, and the appetite had lost its insatiableness, and now to use the old lady's own words, "she could quit eating." Her bowels seldom required the syringe any more.

She was kept upon this treatment, without material variation, for about four weeks, when, so far as the diabetic disease was concerned, an apparent cure, at least, had been effected, and the prescription was discarded. Up to the present writing there has been no return of the original disease, the patient being now able to go abroad, and she has evidently gained considerable flesh. She is using an ordinary mixed diet.

It is proper to state here, that from the first of my seeing the patient in question up to the present time, she has suffered more or less from what seemed to be a simple conjunctivitis. Whether it has any pathological connection with the diabetes is entirely problematical. The eye—the right one only—is still inflamed; and because it was rebellious to treatment, and I had not the power to force it into obedience, the patient became *impatient*, and Dr. B., a quack of the eclectic school, and who had, during almost my entire intercourse with the case, been interfering, was called in. But with the original ailment he has had, nor will he be permitted, I presume, to have anything to do.

Now here is a case of diabetes—mellitus, I suppose—which has been cured, apparently at least, and I have given you the general plan of treatment that was adopted. And here I must not forget to advert to the curability of the disease, as I sometime ago promised to do so. The older writers claim to have cured the disease frequently; but it is to be presumed that many of their cases were not saccharine diabetes, but a sort of vicarious disease. Eberle, a very sagacious physician, says he cured but one case out of six that came under care; and Watson, I believe, supposes the disease to be incurable. But if, as I have supposed, the disease is neurotic in its nature, it must be apparent that it is an affection which may occasionally admit of cure. And, indeed, cures may sometimes take place spontaneously; for if by any means the peculiar irritability of the medula oblongata, upon which it is believed to depend, should be removed, the urine would lose its morbid character, and the patient would recover, provided the system had not been too much depressed to admit of recuperation. How often, for example, do spontaneous cures of epilepsy occur? We have probably all witnessed instances of this kind. So of chorea, and so, also, of paralysis, especially of that variety dependent upon atrophy of the brain.

But why did I employ the nit. of silver in the treatment of the case, brief notes of which I have just read? I might assign several reasons for prescribing the salt of silver. First, I prescribed it because I lacked confidence in any known mode of treatment. Secondly, because, should the essential nature of the disease be an obscure chronic inflammation of the mucous tissue of the stomach and bowels, as many physicians seem to believe, I knew of no remedy better calculated to restore these tissues to a healthy condition than the nit. of silver. Thirdly, if the disease is neurotic, as I have contended it is, then perhaps no agent in the whole catalogue of remedies would seem to promise more good results than this one. Its undoubted efficacy in several forms of nervous disease is well known. Epilepsy, catalepsy and chorea have, it is believed, frequently yielded to the powers of this remedy. But how does nitrate of silver operate in curing certain chronic nervous affections? The only satisfactory answer which I am able to give is that the medicine, as experimentally established, sometimes cures the affections named—and what cured a given case will cure another identically similar case similarly circumstanced. The nitrate of silver is claimed to be tonic, alterative, astringent, etc. It

[For conclusion of Article, see page 661.]

